# Pulmonary Embolism Presenting as an Anterior ST-elevation Myocardial Infarction: A Case Report

**DOI:** 10.5811/cpcem.2020.7.48421

**Published:** 2020-09-23

**Authors:** Varvara Ladage, Miciah Jones, Faheem Ahmad, Cherian Plamoottil, Ryan Misek, Nicole Alexander-Anyaogu

**Affiliations:** * Midwestern University Chicago College of Osteopathic Medicine, Department of Emergency Medicine, Downers Grove, Illinois; † Franciscan Health Olympia Fields, Department of Cardiology, Olympia Fields, Illinois; ‡ Midwestern University Chicago College of Osteopathic Medicine, Department of Clinical Education, Downers Grove, Illinois; § Franciscan Health Olympia Fields, Department of Emergency Medicine, Olympia Fields, Illinois

**Keywords:** Pulmonary embolism, STEMI, chest pain, syncope

## Abstract

**Introduction:**

While the electrocardiogram (ECG) for pulmonary embolism typically shows tachycardia or evidence of right heart strain, it can demonstrate ischemic changes similar to acute coronary syndrome.

**Case Report:**

The patient in this case presented with syncope, chest pain, and an ECG showing an anterior acute myocardial infarction (AMI) without evidence of right heart strain. His cardiac catheterization showed no coronary artery occlusions, but some signs of pulmonary embolism (PE), which was confirmed on computed tomography angiography of the chest.

**Conclusion:**

This case demonstrates that PE should be high on the differential for AMI and describes an uncommonly encountered mimic for classic ST-elevation myocardial infarction ECG changes. Further diagnostics to confirm the diagnosis should be obtained when indicated.

## INTRODUCTION

Acute myocardial infarction (AMI) and pulmonary embolism (PE) can present in similar ways: chest pain; shortness of breath (SOB); and diaphoresis.^1^,^2^ One challenge for AMI presenting with an ST-elevation myocardial infarction (STEMI) is the speed at which patients are supposed to be taken to the catheterization lab.^3^ The current goal for door-to-balloon time is 90 minutes, but studies are looking at potentially shortening these times.^4^ Furthermore, studies that have sought to shorten door-to-balloon time have come with a concomitant increase in negative catheterizations or, non-cardiac, etiologies for the patient’s symptoms.^4^

## CASE REPORT

**Image 1:**
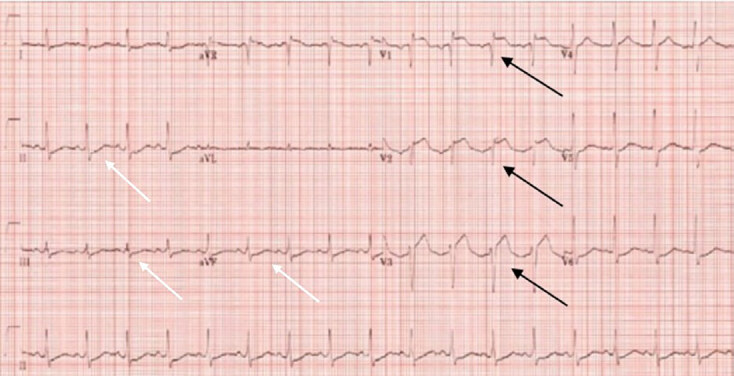
Acute anterior myocardial infarction with ST-segment elevations in V1 through V3 (black arrows) and associated reciprocal changes in leads II, III and aVF (white arrows).

A 56-year-old male with history of multiple myeloma status post-bone marrow transplant on suppressive therapy, hypertension, and peripheral neuropathy presented to the emergency department (ED) after a syncopal episode, where he was found in the bathtub by his family with his eyes rolled back. Upon initial examination, the patient complained of mild chest pain. Upon arrival to the ED, the patient was found to be tachycardic with a pulse of 108 beats per minute, tachypneic with 24 breaths per minute, a blood pressure of 95/68 millimeters mercury, and oxygen saturation of 100% on room air. An electrocardiogram (ECG) demonstrated ST elevations in V1 through V3 with reciprocal changes in the inferior leads (Image 1).

**Image 2:**
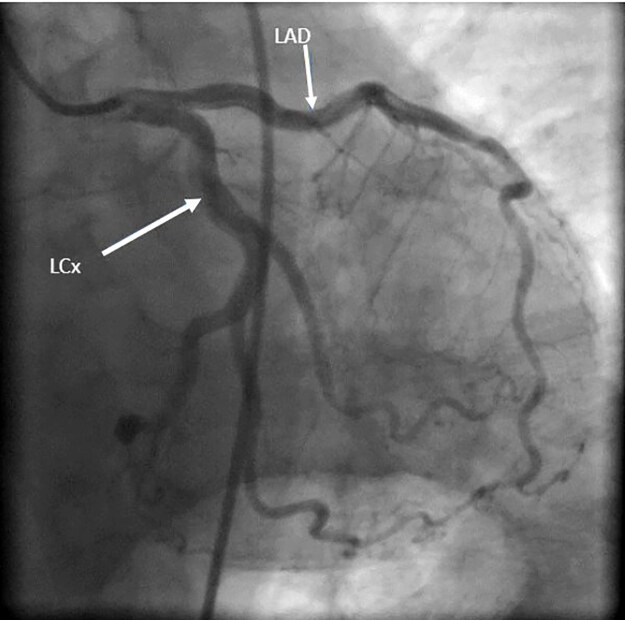
Percutaneous coronary intervention with arrows pointing to the left circumflex (LCx) and left anterior descending (LAD) arteries with no stenosis.

High-sensitivity troponins were obtained and were elevated to 0.20 nanograms (ng) per milliliter (mL) (reference range: normal <0.04 ng/mL). The patient was taken to the cardiac catheterization laboratory where he underwent percutaneous coronary intervention (PCI), which did not reveal severe coronary artery disease (Image 2) but did show hyperdynamic left ventricle with an ejection fraction >70% on ventriculography, tachycardia, and low left ventricular end-diastolic pressure indicating low filling pressures with suspicion for reduced preload. This was concerning for hypovolemia or pulmonary embolus.

**Image 3:**
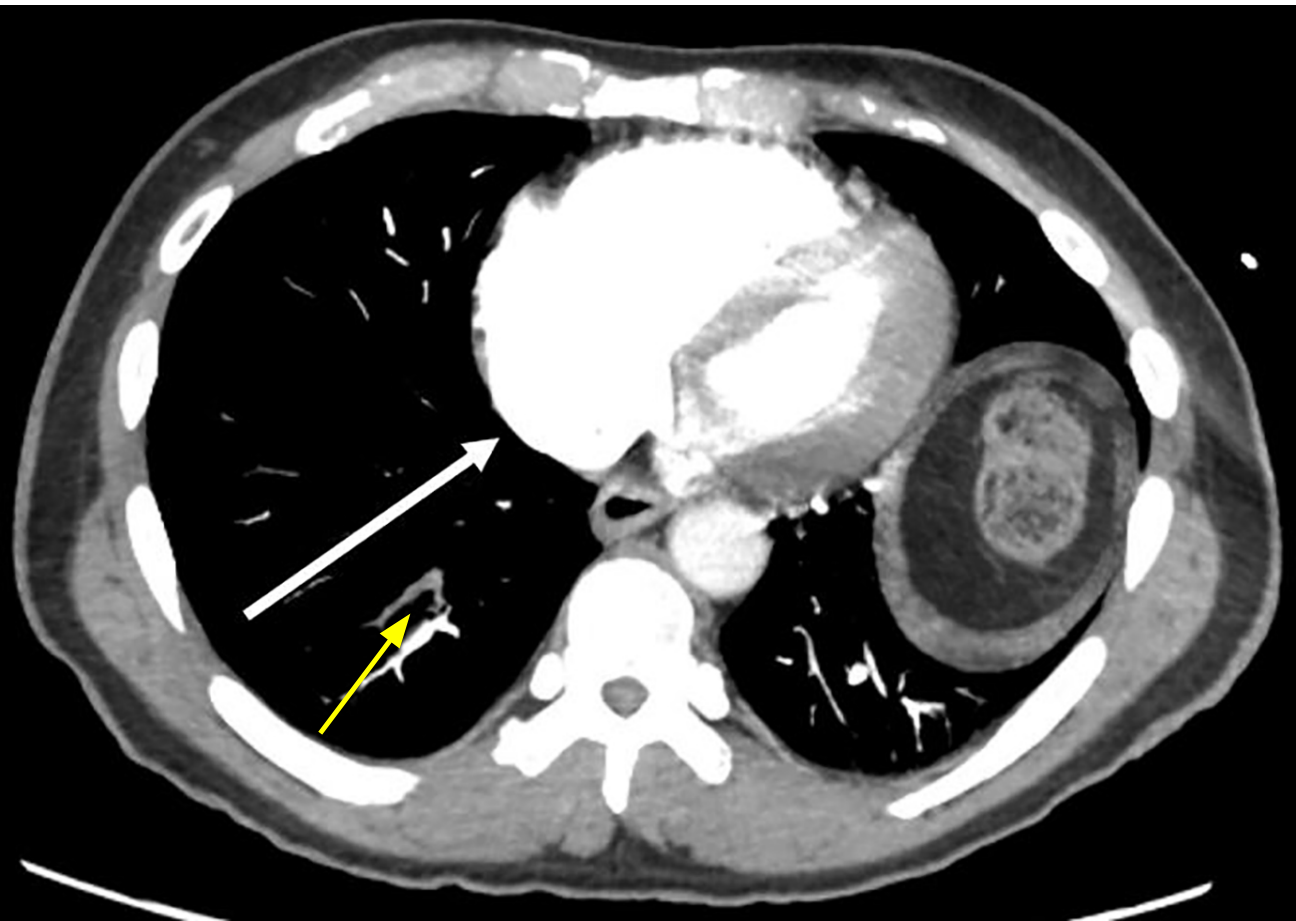
Axial image from computed tomography angiogram of the chest demonstrating significantly dilated right ventricle (arrow) and filling defect consistent with pulmonary embolism (yellow arrow).

Computed tomography angiography (CTA) of the chest was obtained showing submassive PE as well as right ventricular strain with a right ventricle to left ventricle ratio of 1.2 suggestive of cor pulmonale (Image 3).

It was later reported after cardiac catheterization that the patient had had increased bilateral leg swelling and discomfort as well as dyspnea on exertion for several weeks preceding his presentation to the ED, which he attributed to his neuropathy following chemotherapy. The patient was started on a continuous heparin infusion, and repeat ECG demonstrated resolution of anterior ischemic changes. Venous duplex ultrasound of the bilateral lower extremities was obtained on hospital day two and demonstrated a partially occlusive venous thrombus in the left popliteal, peroneal, and posterior tibial veins. The patient underwent catheter-assisted thrombolysis on hospital day three. He was started on apixaban 10 milligrams (mg) twice daily for the first week, followed by 5 mg twice daily, and discharged home in stable condition.

## DISCUSSION

Differentiating between myocardial infarction and PE can be difficult based on initial symptoms alone because they can have similar presentations. PE can present with ST-segment elevations, making it important to consider other conditions that can mimic STEMI.^5^ The patient presented with syncope and dyspnea. Due to time pressures secondary to the door-to-balloon time mandate, his initial history was appropriately brief. Based on the ECG showing ST-segment elevations in leads V1–V3 with reciprocal changes, STEMI was diagnosed and he was taken directly for emergency cardiac catheterization. His elevated troponin lent more evidence to a diagnosis of acute coronary syndrome. When reviewing the ECG in this case, no evidence of right heart strain was present unlike in other cases noted in the literature.^6^–^9^

CPC-EM Capsule

What do we already know about this clinical entity?

*Pulmonary embolisms (PEs) have a variety of presentations, including acute ST-elevation myocardial infarctions (STEMI), chest pain, shortness of breath, and syncope as documented by several case reports in the literature*.

What makes this presentation of disease reportable?

*When PEs present as STEMIs there is typically evidence of right heart strain on electrocardiogram (ECG). In this case, no right heart strain was noted on the ECG*.

What is the major learning point?

*Have pulmonary embolism on your differential diagnosis even if the ECG shows a STEMI with no evidence of right heart strain*.

How might this improve emergency medicine practice?

*The case highlights the importance of having a broad differential for STEMI seen on ECG, including PE, even without any evidence of right heart strain*.

History of syncope, unilateral leg swelling or pain, deep venous thrombosis, sudden dyspnea, recent surgery, or hemoptysis all increase the probability of a PE while the absence of dyspnea, and tachypnea reduce the probability of a PE.^1^ However, no one symptom alone can point to any specific diagnosis. Further testing, such as an ECG, can help narrow the differential when history alone is inadequate. Typically, PEs present as sinus tachycardia on ECG but they can also have ECG findings suggestive of acute right ventricular (RV) strain, such as deep T-wave inversions in leads V1, V2 and V3, complete or incomplete right bundle branch block, or S1Q3T3.^10^ While rare, there have been several documented cases of PE presenting with ST-segment elevation mimicking anterior wall MI.^6^–^9^ This is likely due to acute RV strain and poor left ventricular (LV) filling resulting in decreased blood flow to the coronaries.^11^

Massive PE is defined as a PE with sustained hypotension, need for inotropic support, or persistent bradycardia.^12^ Submassive PEs are typically normotensive but have either myocardial ischemia as evidenced by an elevated troponin or B-type natriuretic peptide, or RV dysfunction visualized typically on ultrasound or an RV to LV ratio of greater than 0.9 on imaging.^12^ Lastly, uncomplicated PEs have no ventricular dysfunction of myocardial ischemia and are normotensive.^12^

In this case, CTA chest demonstrated RV dysfunction, which was not seen on the ECG. The finding of RV dysfunction in patients who present with anterior AMIs on ECG is demonstrated several times in the literature.^6^–^9^ The patient in this case met criteria for a submassive PE due to his normotension, elevated troponin, and visualized RV dysfunction. The RV does not begin to dilate until there is greater than 50–70% occlusion of the pulmonary vasculature.^12^ The dilation of the RV distorts the septum and the LV leading to eventual ventricular desynchrony as well as ST-segment elevations on the ECG.^13^

## CONCLUSION

Since ST-segment elevations are associated with multiple diagnoses such as AMIs, pericarditis, and early repolarization, it is not only important to keep a differential broad but to obtain a thorough history. Clinical history affects the interpretation of ECGs, leading clinicians to search for specific ECG findings or alerting them to other diagnoses that may not have been in the initial differential.^14^ Although emergency physicians have increased time pressure to expedite cardiac catheterization on patients with STEMI on ECG, this pressure can result in increased incorrect activations. While PE presenting as a STEMI is a rare phenomenon, there have been several reported cases.^6^–^9^ Obtaining good initial history, despite mandated time limits, can help further guide the differential to differentiate between PE and STEMI.
